# Efficacy and Safety of Catheter-directed Thrombolysis in Preventing Post-thrombotic Syndrome: A Meta-analysis

**DOI:** 10.7759/cureus.4152

**Published:** 2019-02-28

**Authors:** Luai Alhazmi, Abdelmoneim Moustafa, Muhammad A Mangi, Ahmed Alamer, Ehab Eltahawy

**Affiliations:** 1 Cardiology, University of Toledo Medical Center, Toledo, USA; 2 Internal Medicine, University of Toledo Medical Center, Toledo, USA; 3 Miscellaneous, University of Toledo Medical Center, Toledo, USA

**Keywords:** post-thrombotic syndrome, catheter directed thrombolysis, deep vein thrombosis

## Abstract

Post-thrombotic syndrome (PTS) is a complication that can develop after deep vein thrombosis (DVT) of lower extremities. In this meta-analysis, we compare the different modalities for treatment of DVT in reducing the risk of PTS. The primary outcome was the risk of PTS, and the secondary outcome included the risk of bleeding events. Review Manager (version 5.3; Cochrane Collaboration software) was used to analyze the data that are represented as a forest plot. Meta-analysis indicated that catheter-directed thrombolysis (CDT) plus anticoagulation (AC) decreases the likelihood of developing PTS compared with the AC-only group with an odds ratio of 0.28 (0.12-0.64). A subgroup analysis of randomized control trial (RCT) studies was conducted, and findings suggest a slight decrease in the likelihood of PTS incidence in the CDT+AC treatment group compared to the AC treatment group (odds ratio, OR = 0.76; CI = 0.58-0.99). For the secondary outcome, a statistically significant increase in bleeding events in the intervention groups was reported with an OR of 3.38 (1.33-8.61), suggesting that the risk of bleeding was high in the CDT plus AC group. CDT in addition to conventional AC for patients with DVT decreases the likelihood of PTS development. The protective effect of CDT comes at the expense of an increase in bleeding risk by three-fold. The decision to utilize CDT to prevent PTS should be individualized according to patient risk factors for developing PTS and their risk of bleeding.

## Introduction

Post-thrombotic syndrome (PTS) is a complication that can develop after deep vein thrombosis (DVT) of the lower extremities [1-3]. PTS can occur at various times after the initial episode but usually manifests within two years of initial DVT onset [3].

The clinical presentation of PTS varies in severity from mild, lower extremity swelling to significant complications such as chronic leg pain, venous claudication, and leg ulcerations. PTS is diagnosed primarily based on clinical presentation including signs and symptoms in patients with confirmed DVT. Current recommendations from the American Heart Association (AHA) for clinical diagnosis suggest waiting for acute DVT to subside and then utilizing clinical tools or scales such as the Villalta scale or the clinical, etiological, anatomic, pathophysiological (CEAP) classification among others [2]. PTS can affect the quality of life and functional status of affected patients, while also being a very costly health care burden [2].

A combination of mechanisms contributes to PTS, including venous outflow obstruction, destruction of the venous valvular apparatus, development of venous reflux, calf muscle pump dysfunction, and reduced wall shear stress, which may trigger an inflammatory process within the affected vein. These mechanisms result in elevated venous pressures in the affected limb, particularly in an upright position, and can worsen venous reflux resulting in a vicious cycle of events [1].

Early and more complete thrombus clearance is believed by many physicians to relieve venous outflow obstruction, preserve valvular function, and reduce venous hypertension [3-5]. Systemic thrombolysis showed significant reductions in residual thrombus and PTS development; however, it increased the risk of major bleeding by three folds compared to conventional anticoagulation therapy [6-7]. Consequently, the AHA guidelines recommended against the use of systemic thrombolysis in acute DVT [3]. Catheter-directed thrombolysis (CDT) is an alternative option for decreasing the incidence of PTS with reduction of bleeding events compared to systemic lytic therapy. There is a growing body of evidence supporting the role of CDT in achieving a significant reduction of PTS particularly in patients with DVT by restoring patency of the affected vein, minimizing valvular incompetence and venous hypertension in the early studies [8-9].

In this meta-analysis, CDT along with anticoagulation therapy (CDT+AC) is compared to anticoagulation (AC) therapy alone as treatment options for DVT. The subsequent risk reduction for PTS development following treatment is the primary outcome; the secondary outcome measure is the incidence of a bleeding incident (minor or major).

## Materials and methods

Data sources and search strategy

A systematic review was carried out in accordance with the preferred reporting items for systematic review and meta-analysis (PRISMA) guidelines [10]. An experienced, health sciences reference librarian performed the literature search. Using PubMed, Embase, Cochrane Central Register of Controlled Trials, and Web of Science, published literature was searched from the time frame of database inception until February 25, 2018. Using the options of advanced search, the website was thoroughly filtered for CDT or standard anticoagulation or iliofemoral/popliteal/lower-extremity DVT. To identify further articles, references were hand searched. All identified articles were compiled using Endnote.

Study selection

The eligibility of studies was assessed by two investigators (LA, AM), independently. Studies were included in the present analysis if they were original research (randomized control or case-control studies) and involved patients undergoing therapy for DVT. Therapies being compared must include CDT and AC or AC alone. Studies were included if they measured the outcome variables of interest for this analysis: PTS development and bleeding events.

Studies were excluded if they were case reports or review articles. Studies were also excluded if they did not report the outcomes of interest. Articles published in languages other than English were excluded, only if no translation was available.

Data extraction and quality assessment

Two reviewers (LA, AM) individually extracted data from studies that met the inclusion and exclusion criteria and compiled an electronic database. The extracted information included the year of publication, sample size, the mean age of the patients, the country in which the study took place, PTS diagnosis method, and duration of follow-up. Discrepancies in data extraction and assessing bias in individual studies were resolved through mutual discussion and consensus formulation. The modality for diagnosing PTS differed among included studies, reflecting the unstandardized diagnosis methods in clinical practice, subsequently, various methods, as reported by individual studies, were included in this analysis.

The quality of the selected studies was assessed by two reviewers (LA, HA) using the Newcastle-Ottawa Assessment Scale and a modified Jadad scale for non-randomized, observational studies, and randomized studies, respectively [11-12].

Statistical analysis

Review Manager (version 5.3; Cochrane Collaboration software; Copenhagen) was used for all statistical analysis. Forest plots, illustrating findings were generated. Treatment effects for both primary and secondary outcomes were analyzed using odds ratios, with a two-sided statistical significance level of 5%. Summary statistics were assessed using odds ratios and the statistical heterogeneity of each plot was assessed using I^2^. The level of heterogeneity was demarcated as low (I^2^ = 25% to 49%), moderate (I^2^ = 50% to 74%), and high (I^2^ > 75%) heterogeneity [13]. Forest plots were generated for all studies and subgroup analyses (randomized control trials [RCT], studies utilizing the Villata scale for diagnosing PTS).

## Results

Study selection and characteristics

**Figure 1:**
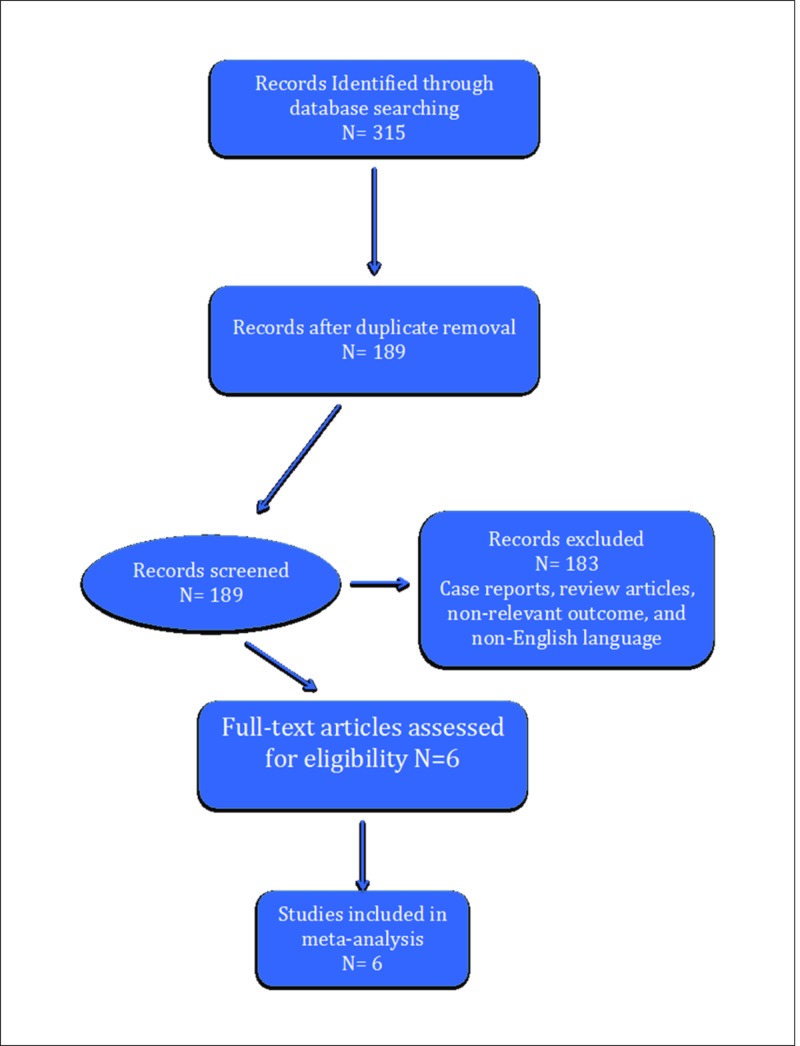
PRISMA flow diagram

A total of 315 papers were identified out of which 126 were duplicates. After further screening, 42 articles were then assessed in full for eligibility. Only six studies were identified after the application of inclusion and exclusion criteria [2-7]. Some studies were excluded due to irrelevant outcomes compared to our meta-analysis [12-13]. The detailed literature search is highlighted in the PRISMA flow diagram (Figure 1).

The participant pool, of all six included studies totaling 1,077 patients, ranging in size from 50 to 692 patients with DVT. The duration of the follow-up ranged from six to 60 months. Two RCTs (CAVENT and ATTRACT trials) and four non-randomized, case-control studies included the experimental groups that received CDT therapy along with AC and control groups that received AC therapy alone, for acute lower extremity DVT [14-19]. Studies included multiple modes of PTS diagnosis including the Villata scale, CEAP classification, and clinical diagnosis based on signs and symptoms [14-15,17-19]. Table 1 outlines the baseline demographic and clinical characteristics of the studies included in analyses.

**Table 1:** Baseline characteristics PTS: post-thrombotic syndrome, CEAP: clinical, etiologic, anatomic, and pathophysiologic, CDT: catheter-directed therapy, AC: anti-coagulation *Clinical diagnosis based on sign and symptoms of PTS

Study	Treatment groups	Sample size (n)	Mean age (years)	Follow-up (months)	Country	PTS Definition
AbuRahma et al. [14]	CDT/AC	18/33	50	55	United States	CEAP classification
Lee et al. [15]	CDT/AC	27/26	59	15	Taiwan	Villalta scale
Srinivas et al. [16]	CDT/AC	27/28	53	6	India	Villalta scale
Ezelsoy et al. [17]	CDT/AC	25/25	NA	14	Turkey	Clinically
Haig et al. [18] (CAVENT trial)	CDT/AC	87/89	50	60	Norway	Villalta scale
Vendantham et al. [19] (ATTRACT trial)	CDT/AC	337/355	53	12	United States	Villalta scale

Primary (incidence of PTS) and secondary (bleeding event) outcome data are shown in Table 2. PTS incidence was the primary outcome variable in all six included studies; only a single study did not include the secondary outcome variable of major or minor bleeding events [14-19].

**Table 2:** Outcome variables: PTS and bleeding event results by study CDT: catheter-directed therapy, AC: anti-coagulation, PTS: post-thrombotic syndrome

Study	Group	PTS	Bleeding
AbuRahma et al. [14]	CDT+AC/AC	4/23	5/5
Lee et al. [15]	CDT+AC/AC	5/13	8/5
Srinivas et al. [16]	CDT+AC/AC	5/19	4/0
Ezelsoy et al. [17]	CDT+AC/AC	7/14	NA/NA
Haig et al. [18] (CAVENT trial)	CDT+AC/AC	37/63	20/0
Vendantham et al. [19] (ATTRACT trial)	CDT+AC/AC	157/171	46/38

Assessment of the quality of the included studies

Quality assessment of the studies included in this meta-analysis showed all the studies to be of good quality. The observational studies being 9/9 on the Newcastle Ottawa scale and the two RCTs with a score of 5/5 on the Jadad scale [14-19].

Primary outcome

**Figure 2:**
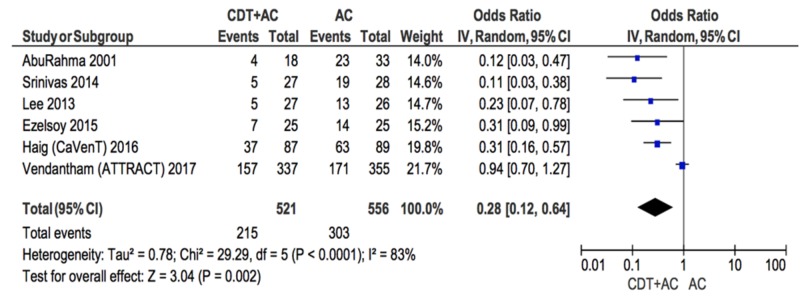
Forest plot comparing PTS incidence in CDT+AC vs AC CDT: catheter-directed therapy, AC: anti-coagulation, PTS: post-thrombotic syndrome

The first forest plot, of all six studies, assessed the treatment effect of CDT+AC therapy compared to AC alone as DVT treatments, in the likelihood of developing PTS. Results indicate that CDT+AC as the therapy for DVT decreases the likelihood of developing PTS compared to AC therapy alone (odds ratio [OR] = 0.28; 95% confidence intervals [95%CI] = 0.12-0.64; Figure 2). The heterogeneity of the included studies was high (I^2 ^= 83%).

The high level of heterogeneity suggested a need to explore sources of heterogeneity. An additional subgroup analysis removed the ATRRACT trial, which reduced the heterogeneity, was to 0%.

Randomized control trials subgroup analysis

**Figure 3:**
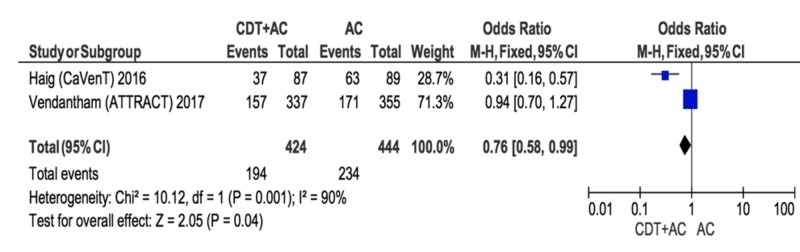
Forest plot comparing PTS incidence in CDT+AC compared to AC in RCTs CDT: Catheter-directed therapy, AC: anti-coagulation, PTS: post-thrombotic syndrome, RCT: randomized control trial

A subgroup analysis of RCT studies was conducted and findings suggest a slight decrease in the likelihood of PTS incidence in the CDT+AC treatment group compared to the AC treatment group (OR = 0.76; [CI] = 0.58-0.99; Figure 3) [18-19]. The heterogeneity of the included studies was high (I^2 ^= 90%).

**Figure 4:**
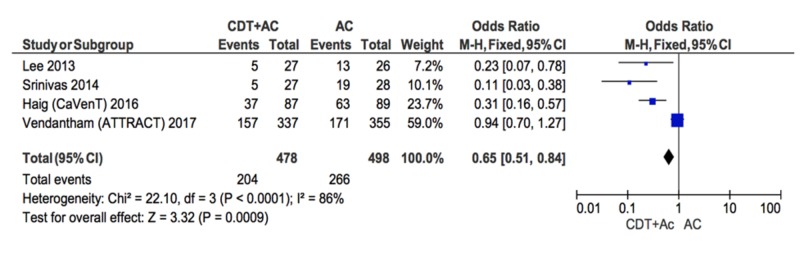
Forest plot comparing PTS in CDT+AC vs. AC in studies that used Villalta scale to define PTS CDT: catheter-directed therapy, AC: anti-coagulation, PTS: post-thrombotic syndrome

Another subgroup analysis included only studies that used the Villalta scale to diagnose PTS (Figure 4) [15,17-19]. Findings suggest the likelihood of PTS development was lower in the group receiving CDT+AC compared to the AC group (OR = 0.65; CI = 0.51-0.84). The heterogeneity of the included studies was high (I^2 ^= 86%).

Secondary outcome

**Figure 5:**
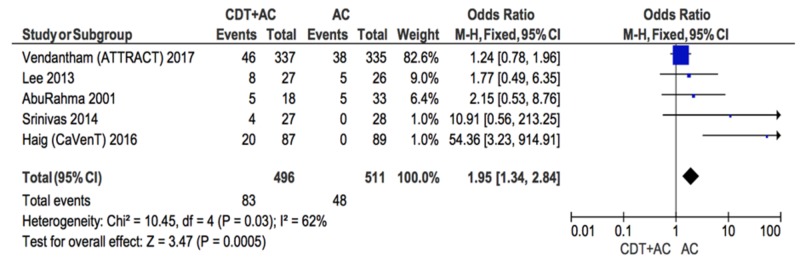
Forest plot comparing the incidence of bleeding events following CDT+AC compared to AC alone CDT: catheter-directed therapy, AC: anti-coagulation

Five of the included studies reported data on major and minor bleeding events. A subgroup analysis included only these five studies (Figure 5). An increased likelihood of bleeding events was found for participants who received CDT+AC compared to those who received AC alone (OR = 1.95; CI = 1.34-2.84). However, no incidence of fatal or intracranial bleeding was reported. The heterogeneity of the included studies was moderate (I^2 ^= 62%).

## Discussion

The present meta-analysis investigated the treatment effects of CDT+AC compared to AC therapy alone, in treating DVT. Specifically, the outcomes of interest were the incidence of the common long-term side effect, PTS as well as major or minor bleeding events. Findings of the analysis suggest that CDT+AC reduced the risk of PTS compared to AC alone. Four of the six of the studies included in the present analysis were non-RCTs; these studies though relatively small in size indicated that CDT+AC was effective in reducing PTS incidence compared to AC alone. The two RCTs included in the present analysis; the CAVENT trial and the ATTRACT trial were larger and designed to more accurately investigate the efficacy of CDT [17-18]. The CAVENT trial was a multicenter, randomized trial in which patients with DVT (Ilio-femoral and popliteal) were assigned to CDT and vitamin K antagonist (VKA) therapy versus VKA therapy. The ATTRACT trial was a multicenter, randomized trial in the United States that used pharmacomechanical catheter-directed thrombolysis (PCDT). The ATTRACT trial had the largest number of participants all studies included in this meta-analysis. The individual findings of this study suggest that there is no significant difference in the likelihood of either PTS development or the occurrence of bleeding events when comparing CDT+AC to AC alone. This differs from the pooled results, which show a decreased likelihood of PTS, this difference could be related to the differences in the follow-up duration of this large study in comparison to some of the smaller studies.

The current meta-analysis demonstrates decreased likelihood in PTS incidence in patients treated with CDT+AC compared to anticoagulation alone. However, heterogeneity of the results of the primary outcome was high and that was predominantly driven by the ATTRACT trial results. The other potential source of heterogeneity between studies relates to differences in pharmacological therapy. Going into the details of these studies, AbuRahma, et al. used urokinase with loading dose 4,500 U/kg followed by infusion of 4,500 U/kg/hour for 24-48 hours [14]. Fibrinogen more than 100 mg/dl and activated partial thromboplastin time twice the control were made as the basis of the treatment. If fibrinogen was less than 100 mg/dl, then urokinase was held for 6-12 hours and additional heparin was given at that time, but when fibrinogen was raised back to more than 100mg/dl, then urokinase resumed. Later, Lee, et al. also used the urokinase as a lytic agent [15]. Urokinase infused 600-1200 U/kg/hour over 48-72 hours with fibrinogen, and aPTT were maintained as the basis of the treatment. Moreover, in another study, streptokinase with a dose of 100,000 U was administered [16]. Two-thirds of the total dose given through catheter and one third through a sheath. The infusion was continued until a satisfactory result achieved. Unlike other studies in our meta-analysis, there was a manual aspiration of thrombus before infusion of lytic agent. Similar to this study, another study using the same mechanism of mechanical removal of thrombus followed by the use of alteplase as a lytic agent gained a successful result [17]. In the CAVENT trial, alteplase was used as a lytic agent with a dose of 0.01 mg/kg/hour, a maximum of 20 mg in 24 hours. The infusion was continued until complete lysis or cease progression of a clot on venography with a maximum duration of 96 hours. In the ATTRACT trial, alteplase <35 mg was infused over 24-30 hours. Regarding the anticoagulation across the studies, coumadin was commonly used as anticoagulation, and goal INR was 2-3. Low molecular weight heparin and heparin were used as a bridge to anticoagulation. Given the nature of different drugs, dosing, and protocol of treatment, different outcomes are published, which created a huge heterogeneity in our meta-analysis.

In an attempt to overcome increased heterogeneity, ATTRACT trial was removed in subgroup analysis and the heterogeneity was reduced to 0 %. The level of heterogeneity in studies included in the meta-analysis indicate a high degree of difference between the studies, suggesting further investigation is warranted.

CDT remains controversial in preventing PTS. The current guidelines recommend AC alone for treatment of proximal DVT with level 2C recommendation [2]. An ongoing RCT (Dutch CAVA trial) comparing CDT to AC alone for treatment of proximal DVT may provide more clinical evidence for the benefit of CDT [20].

Regarding the major and minor bleeding events, the current meta-analysis revealed an increase in the likelihood of bleeding events following CDT+AC therapy compared to AC alone. The major bleeding events included retroperitoneal, gastrointestinal, puncture-site bleeding that required a blood transfusion, bleeding that required surgical intervention, and minor bleeding events including puncture-site bleeding that does not require a blood transfusion, ecchymosis. The CAVENT trial demonstrated a high likelihood of bleeding events following CDT+AC therapy compared to AC therapy. The pooled result demonstrated an increased likelihood of bleeding events but was not as high as the CAVENT alone. This could be related to the difference in duration of follow-up between trials.

Limitations

The current meta-analysis had several limitations; four non-RCTs were compared to two RCTs; differences in these studies might influence the statistical results of pooled analyses. The heterogeneity of included studies was high and can mainly be attributed to the difference in size of studies including in our meta-analysis, mainly the ATTRACT trial, but may also be related to differences in follow-up duration.

## Conclusions

CDT in addition to conventional anticoagulation for patients with DVT decreases the likelihood of PTS development. The protective effect of CDT comes at the expense of an increase in bleeding risk by three-fold. The decision to utilize CDT to prevent PTS should be individualized according to patient risk factors for developing PTS and their risk of bleeding.
